# Meetings of Societies

**Published:** 1894-12

**Authors:** 


### Bristol Medico-Chirurgical Society.

*Annual Meeting, October 10th, 1894.*

Mr. J. Greig Smith, President, in the Chair.

After a vote of thanks to the retiring President (Mr. Greig Smith) had been unanimously passed, Dr. A. J. Harrison took the Chair, and read his inaugural address (see page 273), for which a cordial vote of thanks was given him. The Honorary Secretary (Mr. G. Munro Smith) and the Assistant-Editor and Honorary Librarian (Mr. L. M. Griffiths) read their annual reports, which were adopted. The following officers were chosen for the ensuing year:—President-elect; Mr. Arthur W. Prichard: Honorary Secretary; Mr. J. Paul Bush: Honorary Librarian; Mr. L. M. Griffiths: Members of Committee; Dr. Michell Clarke, Mr. J. Dacre, Mr. W. H. Harsant, Dr. Aust Lawrence, Dr. J. E. Shaw, and Mr. G. Munro Smith.

---

*November 14th, 1894.*

Dr. A. J. Harrison, President, in the Chair.

Mr. C. A. Morton showed a patient with congenital deficiency of junction between ribs and sternum.

Dr. H. Waldo and Dr. Harrison showed patients with lichen planus.

Mr. W. M. Barclay showed a patient with a very useful hand, after complete excision of the wrist for disease following an injury.

Mr. Greig Smith showed two calculi removed by suprapubic operations; a malignant growth removed by pylorectomy; a specimen of resection of malignant stricture of colon; a papillomatous growth in an ovarian cyst; an ovarian cyst showing a large number of secondary growths distributed over the whole of the cyst-wall; and a large uterine fibroid that had caused obstruction by pressure on the rectum.

Dr. J. Michell Clarke read a paper on aortic valve disease, in which ulcerative change supervened, and in which the pulsus bisferiens occurred. Dr. Clarke showed some specimens of hemorrhages in the pons Varolii.

Dr. G. Parker read a paper on chloride of calcium.

---

*December 12th, 1894.*

Dr. A. J. Harrison, President, in the Chair.

Dr. H. Waldo showed (1) a girl, aged 19, with primary lateral sclerosis and resulting spastic paraplegia and deformity; (2) a boy with lichen pilaris.

Mr. Paul Bush showed a man with extensive necrosis of skull.

Mr. C. A. Morton showed a patient with gummata of the skull and limbs.

Mr. A. W. Prichard showed a portion of a catheter which he had removed by means of a supra-pubic operation from the bladder of a man, who had had a calculus removed some years before.

Mr. C. A. Morton reported a fatal case of pyæmia from middle-ear disease, in which the internal jugular vein was ligatured.

Dr. J. Swain read the notes of a successful case of castration for prostatic hypertrophy.

J. Paul Bush, *Hon. Sec.*

